# 气腔扩散阳性的肺腺癌临床病理学特征分析

**DOI:** 10.3779/j.issn.1009-3419.2023.106.18

**Published:** 2023-09-20

**Authors:** Lei FAN, Jilong QIN, Xiaodong LIN, Yue WU, Zhenzhen HE, Ping HE

**Affiliations:** 510120 广州，广州医科大学附属第一医院病理科; Department of Pathology, First Affiliated Hospital of Guangzhou Medical University, Guangzhou 510120, China

**Keywords:** 肺肿瘤, 肺腺癌, 气腔扩散, Lung neoplasms, Pulmonary adenocarcinoma, Spread through air spaces

## Abstract

**背景与目的** 气腔扩散（spread through air spaces, STAS）作为一种新发现的肺癌侵袭方式，其生物学特点及分子特征尚存在争议。本研究旨在探讨STAS与肺浸润性腺癌临床病理学特征及基因改变的关系。**方法** 选取广州医科大学附属第一医院2019年7月至2021年3月确诊的肺非黏液性浸润性腺癌手术切除标本694例，分析STAS与临床病理因素之间的关系。应用免疫组织化学方法检测间变性淋巴瘤激酶（anaplastic lymphoma kinase, ALK）蛋白表达；应用扩增阻滞突变系统-聚合酶链反应（amplification refractory mutation system-polymerase chain reaction, ARMS-PCR）技术检测表皮生长因子受体（epidermal growth factor receptor, EGFR）基因突变；应用反转录-PCR（reverse transcription-PCR, RT-PCR）技术检测肉瘤致癌因子受体（ROS proto-oncogene 1-receptor, ROS1）基因融合。**结果** STAS阳性病例共344例，STAS阴性病例共350例。STAS阳性与肿瘤最大径（P<0.001）、胸膜侵犯（P<0.001）、脉管侵犯（P<0.001）、神经束侵犯（P=0.013）、淋巴结转移（P<0.001）、临床分期（P<0.001）及组织学分型（P<0.001）相关；STAS与ALK蛋白表达（P<0.001）相关。多因素分析表明，STAS阳性与胸膜侵犯（P=0.001）、脉管侵犯（P<0.001）、淋巴结转移（P=0.005）及ALK蛋白表达（P=0.032）相关。**结论** STAS阳性与肺腺癌高侵袭性生物学行为有关，提示不良预后。

## Abstract (en)

**Background and objective** The biological and molecular characteristics of spread through air spaces (STAS), a newly recognized invasive mode of lung cancer, remain controversial. The aim of this study was to investigate the clinicopathological features and molecular characteristics of STAS in patients with pulmonary adenocarcinoma. **Methods ** A total of 694 resected invasive non-mucinous lung adenocarcinomas diagnosed by clinicopathology from July 2019 to March 2021 in the First Affiliated Hospital of Guangzhou Medical University were collected, and the relationship between STAS and clinicopathological factors was analyzed. The state of protein expression of anaplastic lymphoma kinase (ALK) was detected by immunohistochemical method. Epidermal growth factor receptor (EGFR) was detected by amplification refractory mutation system-polymerase chain reaction (ARMS-PCR). ROS proto-oncogene 1-receptor (ROS1) was detected by reverse transcription-PCR (RT-PCR). **Results** A total of 344 STAS positive cases and 350 STAS negative cases were collected. By univariate analysis, STAS positivity was statistically associated with tumor maximum diameter (P<0.001), pleural invasion (P<0.001), lymphovascular invasion (P<0.001), nerve invasion (P=0.013), lymph node metastasis (P<0.001), clinical stage (P<0.001) and histological type (P<0.001). There was a statistical correlation between STAS and ALK protein expression (P=0.001). Multivariate analysis showed that STAS positive was correlated with pleural invasion (P=0.001), vascular invasion (P<0.001), lymph node metastasis (P=0.005)and ALK protein expression (P=0.032). **Conclusion ** STAS is associated with highly aggressive biological behavior of lung adenocarcinoma, suggesting a poor prognosis.

肺癌是目前世界上死亡率最高的肿瘤^[1]^，也是中国最常见的癌症^[2]^。近年来肺腺癌的发病率不断攀升，已经超过鳞癌成为肺癌最常见的组织学亚型。尽管通过影像学筛查，肺癌的早期发现率已大幅提升，但仍有较大比例的早期肺腺癌患者在手术后会出现复发和转移^[3]^。由此可见，预测早期肺腺癌的复发风险以及研究肺腺癌的侵袭、转移机制尤为重要。

近年来，病理学家发现除传统观念的肺腺癌浸润方式（间质、脉管及胸膜侵犯）外，肿瘤细胞呈巢团状或团簇状在肺泡腔内进行播散的现象同样具有侵袭作用，2021版世界卫生组织（World Health Organization, WHO）肺肿瘤病理分类将该现象命名为气腔扩散（spread through air spaces, STAS）（图1A-图1D）。

**图1:**
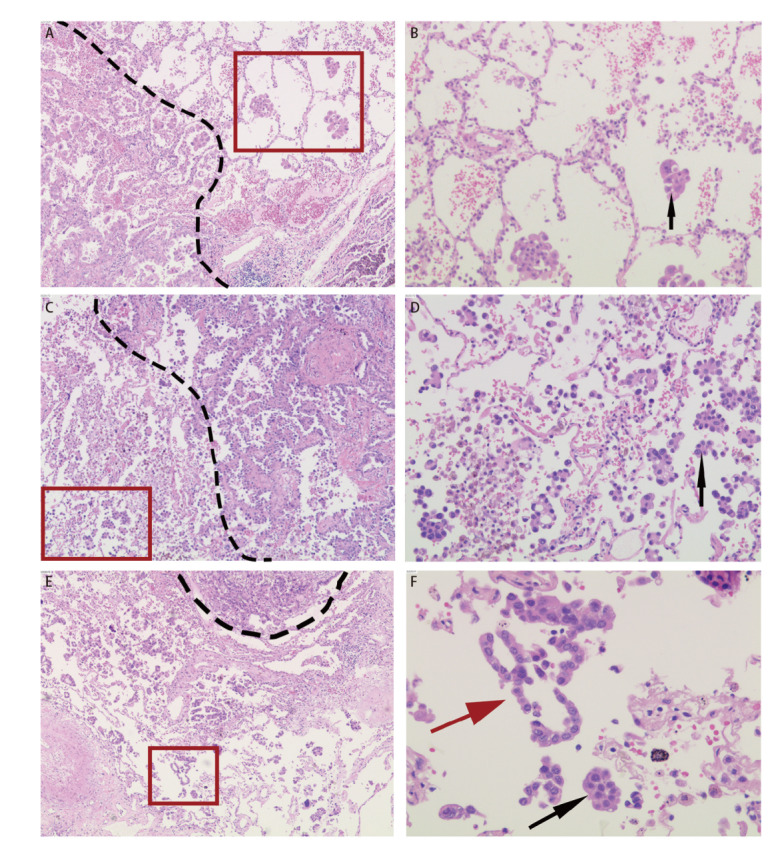
肺浸润性腺癌气腔扩散及人为假象图像（HE：A、C、E：×40；B、D：×100；F：×200）。A：浸润性腺癌（虚线以左为肿瘤主体）；B：STAS现象；C：浸润性腺癌（虚线以右为肿瘤主体）；D：STAS现象；E：浸润性腺癌（虚线以上为肿瘤主体）；F：STAS现象及人为假象（黑色箭头示STAS肿瘤细胞簇；红色箭头示线条状肿瘤细胞）。虚线：肿瘤边界；红色方框：STAS。

STAS独特的形态学表现及生物学特性，吸引了众多学者的关注。有多项研究^[4⇓⇓⇓-8]^显示STAS可作为肺癌不良预后的独立危险因素，因此，STAS的精确诊断有助于为患者制定更合适的手术方案及治疗策略。但目前由于STAS的判读标准未能完全统一，STAS基因状态的研究结果尚不完全一致，对于STAS究竟是病理制片过程中的人为假象还是肿瘤侵袭的一种表现形式尚存在争议，所以有必要对STAS的生物学行为及基因状态进行深入研究。本文拟对STAS与肺腺癌的临床病理学特征及分子改变之间的相关性进行探讨，以进一步为STAS的研究提供依据。

## 1 资料与方法

### 1.1 资料收集

纳入2019年7月至2021年3月于广州医科大学第一附属医院行肺癌根治术的肺非黏液性浸润性腺癌共3347例，其中STAS阳性病例为344例。综合评估后，通过倾向性评分匹配方法，选取同时间段内STAS阴性病例350例。记录患者年龄、性别及病理学特征等。此研究已通过广州医科大学附属第一医院伦理委员会审批（No.2020第K-55），患者及家属均知情同意并自愿参加。

### 1.2 病理诊断方法

肺腺癌诊断标准：结合手术切除标本及病理检查结果，肿瘤病理学以肿瘤原发灶-淋巴结-转移（tumor-node-metastasis, TNM）分期为准，依据为第八版美国癌症联合会（American Joint Committee on Cancer, AJCC）癌症分期手册。肺腺癌标本按照2021年WHO胸部肿瘤分类进行组织学分型，并对其生长方式及有无胸膜、脉管、神经束侵犯及淋巴结转移等临床病理学特征进行分析。

STAS诊断标准：主瘤体边界外的气腔内出现微乳头状细胞簇、实性细胞巢或单个肿瘤细胞。本研究中将超过肿瘤主体3个肺泡腔且存在于肺组织内的肿瘤细胞判定为STAS现象。无法区分肿瘤细胞与组织细胞时，采用免疫组化细胞角蛋白7（cytokeratin 7, CK7）/甲状腺转录因子1（thyriod transcription factor-1, TTF-1）协助诊断。

人为假象的判定：（1）肺泡腔内随机出现的单个细胞，且缺乏持续扩散现象；（2）沿肺泡壁脱落的线性细胞带及锯齿状细胞团簇（图1E，图1F）；（3）位于组织边缘或切片水平面以外的肿瘤细胞簇；（4）正常的良性肺细胞或支气管细胞；（5）与主瘤体组织学形态不一的肿瘤细胞。以上情况均不纳入样本统计。

胸膜侵犯诊断标准^[9]^：胸膜侵犯分为PL0-3级。PL0为肿瘤细胞紧邻脏层胸膜但未超出外弹力板；PL1为肿瘤细胞超出外弹力板，但未突破脏层胸膜间皮层；PL2为肿瘤细胞突破脏层胸膜间皮层，但未侵犯壁层胸膜；PL3为侵犯壁层胸膜。本研究中将PL1-3均判定为胸膜侵犯。

间变性淋巴瘤激酶（anaplastic lymphoma kinase, ALK）蛋白表达阳性免疫组化判读标准如下：以肿瘤细胞胞浆弥漫性、强颗粒状着色判读为蛋白表达阳性（图2）。胞膜着色则为阴性。

**图2:**
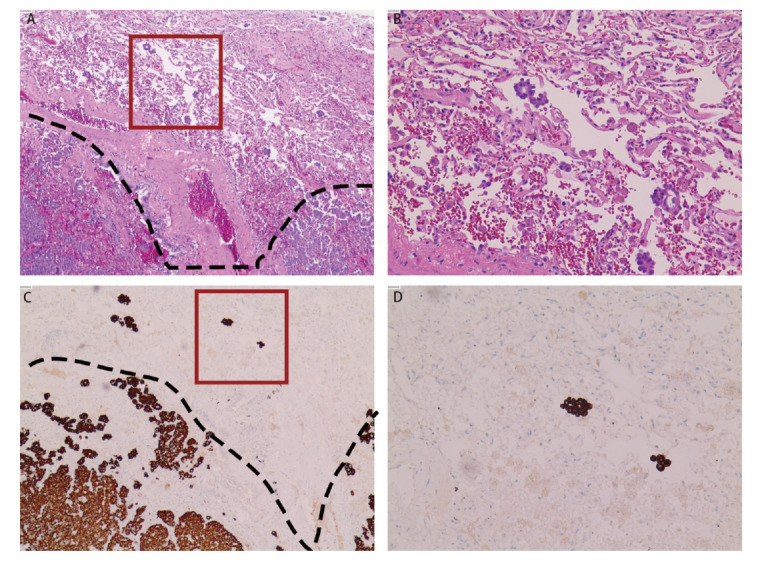
120 mm STAS的HE染色及ALK免疫组化染色（HE：A：×40，B：×200；ALK免疫组化染色：C：×40，D：×200）。A、C：浸润性腺癌（虚线以下为肿瘤主体）；虚线：肿瘤边界；红色方框：STAS；B、D：STAS现象。

### 1.3 质量控制

病理阅片由两名高年资呼吸病理亚专科医生采取双盲法完成，排除意见不一致的病例。为避免现患新发病例偏倚，均选用2019年7月至2021年3月首次诊断的新发病例。

### 1.4 免疫组化

标本均经10%中性福尔马林固定，石蜡包埋，3 μm厚切片，行苏木素-伊红（hematoxylin-eosin, HE）及免疫组化染色。间变性淋巴瘤激酶[ALK（D5F3）]伴随诊断试剂盒（Ventana即用）、OptiView DAB免疫组化检测试剂盒均购自瑞士罗氏公司。免疫组化染色均设立阴性及阳性对照，其中阴性对照采用兔单克隆阴性质控抗体。检测设备为罗氏全自动免疫组化染色仪（型号：BenchMark ULTRA），染色流程按照试剂盒说明书进行。

### 1.5 基因检测

表皮生长因子受体（epidermal growth factor receptor, EGFR）基因突变检测采用扩增阻滞突变系统-聚合酶链反应（amplification refractory mutation system-polymerase chain reaction, ARMS-PCR）技术，肉瘤致癌因子受体（ROS proto-oncogene 1-receptor, ROS1）基因融合检测采用反转录-PCR（reverse transcription-PCR, RT-PCR）技术。

EGFR基因突变检测试剂盒、ROS1融合基因检测试剂盒及福尔马林固定石蜡包埋（formalin-fixed and parrffin-embedded, FFPE）DNA/RNA核酸提取试剂盒均购自厦门艾德生物医药科技有限公司。MX3000P实时荧光定量PCR仪购自安捷伦科技（中国）有限公司。具体检测流程均依据操作指南及试剂盒说明书进行。

### 1.6 数据分析

采用SPSS 26.0软件进行数据的统计分析。符合正态分布的定量资料以均数±标准差表示；计数资料采用例数（百分比）描述，采用χ^2^检验、χ^2^检验校正公式或Fisher确切概率法进行分析；采用二分类Logistic回归模式进行多因素分析。P<0.05为差异具有统计学意义。

## 2 结果及分析

### 2.1 临床病理学资料

研究人群的临床病理特征见表1。694例肺腺癌患者中，男性336例（48.4%），女性358例（51.6%），其中年龄>65岁者为215例（31.0%），≤65岁者为479例（69.0%），年龄范围为25.00-85.00岁，平均年龄为（59.76±8.33）岁。肿瘤最大径范围为0.60-8.50 cm，平均值为（2.38±1.24） cm，其中543例（78.2%）肿瘤最大径≤3 cm，151例（21.8%）肿瘤最大径>3 cm。肿瘤分期中，I期为475例（68.4%），II期为81例（11.7%），III期为113例（16.3%），IV期为25例（3.6%）。

**表1:** STAS与临床病理因素的分析

Variables	All (n=694)	STAS		P
		Negative (n=350)	Positive (n=344)	
Gender				0.258
Male	336 (48.4%)	162 (46.3%)	174 (50.6%)	
Female	358 (51.6%)	188 (53.7%)	170 (49.4%)	
Age (yr)				0.360
≤65	479 (69.0%)	236 (67.4%)	243 (70.6%)	
>65	215 (31.0%)	114 (32.6%)	101 (29.4%)	
Tumor size（cm）				<0.001
≤3	543 (78.2%)	296 (84.6%)	247 (71.8%)	
>3	151 (21.8%)	54 (15.4%)	97 (28.2%)	
Pleural invasion				<0.001
Negative	486 (70.0%)	299 (85.4%)	187 (54.4%)	
Positive	208 (30.0%)	51 (14.6%)	157 (45.6%)	
Lymphovascular invasion				<0.001
Negative	447 (64.4%)	295 (84.3%)	152 (44.2%)	
Positive	247 (35.6%)	55 (15.7%)	192 (55.8%)	
Nerve invasion				0.013
Negative	673 (97.0%)	345 (98.6%)	328 (95.3%)	
Positive	21 (3.0%)	5 (1.4%)	16 (4.7%)	
Nodal metastasis				<0.001
Negative	539 (77.7%)	310 (88.6%)	229 (66.6%)	
Positive	155 (22.3%)	40 (11.4%)	115 (33.4%)	
Clinical stage				<0.001
I	475 (68.4%)	295 (84.3%)	180 (52.3%)	
II	81 (11.7%)	22 (6.3%)	59 (17.2%)	
III	113 (16.3%)	30 (8.6%)	83 (24.1%)	
IV	25 (3.6%)	3 (0.8%)	22 (6.4%)	
Histological subtype				<0.001
Acinar	411 (59.2%)	215 (61.4%)	196 (57.0%)	
Solid	50 (7.2%)	18 (5.2%)	32 (9.3%)	
Papillary	106 (15.3%)	42 (12.0%)	64 (18.6%)	
Micropapillary	43 (6.2%)	4 (1.1%)	39 (11.3%)	
Lepidic	76 (11.0%)	70 (20.0%)	6 (1.7%)	
Complex glandular	8 (1.2%)	1 (0.3%)	7 (2.1%)	

生物学行为特征：胸膜侵犯208例（30.0%），脉管侵犯247例（35.6%），神经束侵犯21例（3.0%），淋巴结转移155例（22.3%）。组织病理学亚型特征：腺泡型411例（59.2%），乳头型106例（15.3%），贴壁型76例（11.0%），实性50例（7.2%），微乳头型43例（6.2%），复杂腺体型8例（1.2%）。

STAS阳性病例中，ALK阳性48例（14.4%, 48/333），ROS1阳性4例（2.2%, 4/183），EGFR阳性106例（57.9%, 106/183）。STAS阴性病例中，ALK阳性12例（4.2%, 12/287），ROS1阳性3例（1.8%, 3/164），EGFR阳性101例（61.6%, 101/164）（表2）。

**表2:** STAS与ALK蛋白表达及ROS1融合、EGFR突变的分析

Variables	All	STAS		P
		Negative	Positive	
ALK (n=620)				<0.001
Negative	560 (90.3%)	275 (95.8%)	285 (85.6%)	
Positive	60 (9.7%)	12 (4.2%)	48 (14.4%)	
ROS1 (n=347)				0.814
Negative	340 (98.0%)	161 (98.2%)	179 (97.8%)	
Positive	7 (2.0%)	3 (1.8%)	4 (2.2%)	
EGFR (n=347)				0.488
Negative	140 (40.3%)	63 (38.4%)	77 (42.1%)	
Positive	207 (59.7%)	101 (61.6%)	106 (57.9%)	

ROS1: ROS proto-oncogene 1-receptor; EGFR: epidermal growth factor receptor.

所有检测病例中EGFR突变阳性共207例，EGFR基因突变率为59.7%（207/347），在STAS阳性及阴性病例中EGFR突变率分别为57.9%与61.6%。单位点突变前5位分别是：L858R有93例（44.9%, 93/207），19-Del有78例（37.7%, 78/207），G719X有10例（4.8%, 10/207），L861有4例（1.9%, 4/207），S768I有4例（1.9%, 4/207）。STAS阳性病例中，单位点突变前五位分别是：L858R有42例（39.6%, 42/106），19-Del有41例（38.7%, 41/106），G719X有6例（5.7%, 6/106），L861有3例（2.8%, 3/106），S768I有3例（2.8%, 3/106）。

### 2.2 统计学分析结果

单因素分析显示，STAS阳性与肿瘤最大径（P<0.001）、胸膜侵犯（P<0.001）、脉管侵犯（P<0.001）、神经束侵犯（P=0.013）、淋巴结转移（P<0.001）、临床分期（P<0.001）、组织学分型（P<0.001）（表1）及ALK蛋白表达（P<0.001）（表2）相关；与性别（P=0.258）、年龄（P=0.360）（表1）、ROS1融合（P=0.814）及EGFR突变（P=0.488）（表2）无关。

多因素分析显示，胸膜侵犯（P=0.001）、脉管侵犯（P<0.001）、淋巴结转移（P=0.005）及ALK蛋白表达（P=0.032）与STAS阳性相关（表3）。此外，临床分期和组织学分型与STAS阳性有关。在临床分期中，以I期作为参照，II、III、IV期发生STAS的可能性分别是4.354、3.642及6.125倍。组织学分型中，以贴壁型生长方式为主的肺腺癌为参照，微乳头型生长方式为主的肺腺癌发生STAS阳性的可能性是34.503倍，复杂腺体结构为主是21.153倍，其余乳头型、实体型及腺泡型分别为8.982、7.096及5.235倍。

**表3:** STAS与相关病理因素多因素回归分析

Variables	OR	95%CI	P
Age	1.135	0.665-1.938	0.642
Tumor size	1.049	0.546-2.013	0.887
Gender	1.092	0.650-1.833	0.739
Pleural invasion	0.370	0.205-0.667	0.001
Lymphovascular invasion	0.171	0.093-0.315	<0.001
Nerve invasion	1.638	0.302-8.884	0.567
Nodal metastasis	0.339	0.158-0.726	0.005
ALK	0.246	0.068-0.887	0.032
EGFR	0.631	0.110-3.638	0.607
ROS1	1.090	0.628-1.891	0.760
Clinical stage			
I	Reference		
II	4.354	2.034-9.320	<0.001
III	3.642	1.384-9.587	0.009
IV	6.125	1.573-23.854	0.009
Histological subtype			
Lepidic	Reference		
Solid	7.096	2.377-21.184	<0.001
Papillary	8.982	3.474-23.227	<0.001
Micropapillary	34.503	8.648-137.657	<0.001
Complex glandular	21.153	2.051-218.169	0.010
Acinar	5.235	2.171-12.626	<0.001

OR: odd ratio; CI: confidence interval.

## 3 讨论

STAS在2021版WHO肺肿瘤病理分类中被定义为“出现在主瘤体边界外肺泡腔内的微乳头状肿瘤细胞簇、实性肿瘤细胞巢或单个肿瘤细胞”。STAS的提出意味着肿瘤对“空气”的侵袭作为一个新的概念被普遍接受，除传统的肺腺癌浸润方式（间质、脉管或胸膜侵犯）外，STAS被确定为第4种肺浸润性腺癌的侵袭模式。

既往在STAS分级研究中，主要是从STAS肿瘤细胞簇与主瘤体之间的距离及STAS肿瘤细胞簇的数量两个方面进行分析。Warth等学者^[10]^以STAS肿瘤细胞簇与肿瘤主体之间的距离作为判定标准，将距离小于3个肺泡者定义为局限性STAS，将距离大于3个肺泡者定义为广泛性STAS，研究结果提示3个肺泡腔以上的广泛STAS现象更易出现在以高级别生长方式为主的肺腺癌中。另Han的团队^[5]^在研究中以2500 μm（1个×10物镜的视野）作为分界点，将距离<2500 μm定义为STAS I级，距离≥2500 μm定义为STAS II级，该研究提示具有STAS II级的肺腺癌预后更差。Toyokawa等^[11]^以STAS肿瘤细胞簇的数量作为研究对象，根据肿瘤细胞数量将STAS分为3类：“no STAS”（未见明确肿瘤细胞）、“low STAS”（1-4个单个或簇状肿瘤细胞）及“high STAS”（5个及5个以上单个或簇状肿瘤细胞），结果显示肿瘤细胞数量的多少与不良预后之间具有正相关关系。以上文献研究表明，当STAS距肿瘤主体超过3个肺泡腔以及STAS数量≥5个肿瘤细胞时更具有侵袭性，故本研究将肿瘤细胞距离主瘤体≥3个肺泡腔作为STAS阳性的判定标准。

本研究中，STAS阳性病例有192例（55.8%）出现脉管侵犯，157例（45.6%）出现胸膜侵犯，115例（33.4%）出现淋巴结转移。经多因素回归分析，胸膜侵犯（P=0.001）、脉管侵犯（P<0.001）及淋巴结转移（P=0.005）与STAS有关。结果表明STAS阳性病例发生胸膜侵犯、脉管侵犯及淋巴结转移的风险更大，提示预后更差。

此前有研究^[8,10,12,13]^显示高级别生长方式为主的肺腺癌中更易出现STAS，且多以广泛性STAS为主。本研究中STAS在不同病理亚型肺腺癌中出现的比例为：腺泡型47.7%（196/411）、实体型64.0%（32/50）、乳头型60.4%（64/106）、微乳头型90.7%（39/43）、贴壁型7.9%（6/76）、复杂腺体型87.5%（7/8）。统计数据表明，以微乳头型生长方式为主的肺腺癌中最容易出现STAS阳性，其余依次为复杂腺体型、乳头型及实体型肺腺癌（表3）。

迄今，不同研究小组对STAS的分子学改变进行了研究，并展示了相应的结果。有研究^[14]^表明STAS阳性的患者大多容易出现EGFR（-）、KRAS（-）、BRAF（-）、ALK（+）和HER2野生型这5种分子学改变。STAS与野生型EGFR基因状态相关^[10,14⇓⇓⇓⇓-19]^。STAS在肺腺癌中与ALK和ROS1蛋白表达有关^[20]^，Tian等^[15]^及Sun等^[16]^也证实了在具有ALK重排的肺腺癌中更易观察到STAS现象。

本研究中STAS阳性与ALK蛋白表达有关，但与EGFR突变及ROS1融合无明显关系。统计结果示STAS阳性病例中ALK蛋白阳性率为14.4%（48/333），STAS阴性病例中ALK蛋白阳性率为4.2%（12/287）。STAS阳性病例的ALK蛋白阳性率远高于STAS阴性患者。研究结果与Gao等^[21]^及Shin等^[22]^学者的观点一致。本研究中ROS1阳性率在STAS阳性及阴性人群中均不高，分别为2.2%及1.8%。STAS与提示患者预后不良的高危因素及ALK蛋白表达均有关，故我们推测ALK阳性肺癌的不良预后可能与STAS的出现有关。

本研究中，EGFR突变位点主要为L858R（44.9%）及19-Del（37.7%），总占比可达EGFR突变阳性病例的82.9%。但本研究在STAS阳性及STAS阴性组间未发现EGFR突变率及突变位点的差异。

本研究存在不足之处，因研究对象均为2019年7月之后的病例，随访时间较短，故未能进行生存及预后分析。另外，本研究未能按照STAS细胞群的位置、大小、数量不同对病例进行分层分析。

本研究结果显示STAS阳性与肺癌肿瘤大小、胸膜侵犯、脉管侵犯、神经束侵犯、淋巴结转移及临床分期有关，表明STAS并非病理制片过程中的人为假象，而确实是肿瘤侵袭的一种表现形式。在临床工作中，STAS的判断能为临床术式选择、精准治疗及预后判断等方面提供有价值的信息，病理医师应当重视对于肺腺癌中STAS的识别和诊断。

Competing interests

The authors declare that they have no competing interests.

Author contributions

He P conceived and designed the study. Fan L performed the experiments and analyzed the data. Qin JL contributed analysis tools. Lin XD contributed immunohistochemical techniques. Wu Y and He ZZ collected the data. He P provided critical inputs on design, analysis, and interpretation of the study. All the authors had access to the data. All authors read and approved the ﬁnal manuscript as submitted.
